# The Human-Animal Relationship in Australian Caged Laying Hens

**DOI:** 10.3390/ani9050211

**Published:** 2019-05-02

**Authors:** Lauren E. Edwards, Grahame J. Coleman, Kym L. Butler, Paul H. Hemsworth

**Affiliations:** 1 Animal Welfare Science Centre, Faculty of Veterinary and Agricultural Sciences, University of Melbourne, Parkville, Victoria 3010, Australia; grahame.coleman@unimelb.edu.au (G.J.C.); kym.butler@unimelb.edu.au (K.L.B.); phh@unimelb.edu.au (P.H.H.); 2 Department of Jobs, Precincts and Regions, Agriculture Research Victoria, Hamilton, Victoria 3300, Australia

**Keywords:** human-animal relationship, fear, laying hen, stockpeople attitudes, stockperson behaviour, egg farm, albumen corticosterone, welfare

## Abstract

**Simple Summary:**

Stockperson behaviour can influence fear of humans and welfare of farm animals. This study observed the human-animal relationship (HAR) in 19 Australian caged laying hen flocks to determine whether stockperson behaviour was associated with behavioural indicators of fear of humans and stress in caged laying hens. The average avoidance response of each flock toward an approaching human was assessed using two behavioural tests, and stress was measured using the concentration of corticosterone in an egg sample collected immediately prior to these observations. Stockperson behaviour was observed for 2 days in each flock and compared to hen fear. Unexpectedly, no relationships were found between the observed stockperson behaviour and avoidance of humans in the hens, but flocks were more productive when they showed less avoidance of humans, and when stockpeople made less noise in the laying house. This suggests that stockperson behaviour and hen fear may influence productivity, but there was no evidence that any effect of fear on productivity was caused by stockperson behaviour. Unexpectedly, the most fearful flocks also had the lowest stress levels. These results clearly need further research to be fully understood but could not confirm the existence of the HAR on caged egg farms in Australia.

**Abstract:**

Studies on farm animals have shown relationships between stockperson attitudes and behaviour and farm animal fear, stress and productivity. This study investigated how the avoidance behaviour of Australian commercial caged laying hens may be related to stockperson behaviour, albumen corticosterone, and the number of weeks producing within 5% of peak egg production. Nineteen laying houses were assessed over 3 days. Fear of humans in hens, based on their avoidance response to an unfamiliar human, was assessed using two behavioural tests. Albumen corticosterone concentrations were measured from egg samples collected immediately prior to behavioural testing. Stockperson attitudes were assessed using a questionnaire and stockperson behaviour was observed over 2 days. Productivity records for each laying house were also obtained. The duration of peak production was negatively related to both noise made by the stockperson and hen avoidance. No relationship between stockperson behaviour or attitudes and hen avoidance was found, but stockpeople with negative attitudes made more noise. In conclusion, this study could not confirm a relationship between stockperson behaviour and hen avoidance behaviour for Australian caged laying hens. However, this study did confirm a relationship between hen avoidance behaviour, albumen corticosterone concentration, and the duration of peak egg production.

## 1. Introduction

There is increasing evidence that the interactions between stockpeople and their animals can have a substantial effect on the behaviour, welfare and productivity of farm animals by causing fear of humans and fear-related stress in these animals [1,2]. The nature and extent of these human-animal interactions will determine the quality of the human-animal relationship (HAR), and are determined by stockperson attitudes towards their animals and their work, their beliefs about other people’s expectations of them, and their beliefs about the extent to which they have control over their ability to appropriately interact with the animals [1,2]. For example, the frequent use of aversive behaviours by stockpeople increases fear of humans in both sows [3] and dairy cattle [4,5,6], and the reduced productivity observed in these animals has been attributed to fear-induced stress [1].

Similar relationships have also been demonstrated in laying hens. Under experimental conditions, Barnett et al. (1994) [7] demonstrated that exposing cage-housed hens to additional human contact of a positive nature reduced their fear of humans, based on their avoidance behaviour in an approaching human test and corticosterone response to handling. The additional human contact also improved their immune function, based on a cell-mediated response following injection with a mitogen, and hen day production in comparison to hens that received minimal human contact. Under commercial free-range conditions, Waiblinger et al. (2018) [8] found significant relationships between stockperson attitudes, their subsequent interactions with the laying hens, the number of hens that could be approached and touched in the flock, and the feather damage and mortality rates of those hens. Stockpeople with positive attitudes toward hen care had flocks with lower avoidance behaviour, while stockpeople with negative general attitudes had flocks with more feather damage and higher mortality. Several other studies have shown relationships between human behaviour and fear of humans in hens [9,10,11], and between fear of humans and hen productivity [12]. These studies suggest that stockperson behaviour may be an important determinant of the avoidance behaviour, stress physiology and productivity of commercial laying hens.

This paper investigates how one aspect of hen productivity, namely the number of production weeks within 5% of peak egg production, may, through fear of humans, be related to attitudes and behaviours of stockpersons in commercial cage-housing systems within Australia.

## 2. Materials and Methods

This research had ethics approval from the Department of Primary Industries Animal Ethics Committee (AEC Code No. 2627) and the Melbourne School of Land and Environment Human Ethics Advisory Group (HREC Project No. 050041).

### 2.1. Subjects, Location and Summary of Data Collection

Data were collected at 19 laying houses from 10 farms, representative of commercial caged egg enterprises that were located in the states of Victoria or New South Wales, Australia. The study was conducted over 15 months. A summary of the laying houses visited is presented in Table 1. Each laying house was considered a separate unit if a separate team of stockpeople maintained it, and thus more than one laying house could be studied at the same farm. Multiple houses were studied at four farms. In each laying house a sample of focal cages was selected for closer study.

Where possible, the productivity records for the entire laying life of the flock in each study house were obtained. Data were collected at each laying house over a 3-day period, with 1 day spent conducting avoidance behaviour tests on the hens and the following 2 days spent observing the stockperson behaviour that occurred in the laying house. At the end of the third day a written attitude questionnaire was administered to the main stockperson that worked in each house.

### 2.2. Physical Features of the Laying House

The following measurements were made of the laying houses: the total number of stockpeople that entered the laying house (No. of Stockpeople); the age of the flock in weeks (Age of Birds); the length of the aisle in m (Aisle Length); the height, depth and width of the cages in cm (Cage Height, Cage Depth, Cage Width); the average number of birds in each focal cage (Av. No. of Birds per Cage); the number of birds in the laying house (Flock Size); the average space allowance in cm^2^ per bird in each focal cage (Space Allowance), the light level at the door of each focal cage in lux (Lux) and the strain of laying hen used (Strain). Lux was logarithmically transformed before being used in statistical analysis.

It should be noted that the conventional cages used for laying hens in Australia do not contain enrichments such as perches or nest boxes. Legislation exists in regard to the minimum space allowance (550 cm^2^/hen) and certain aspects of cage design (e.g., floor slope ≤8°, 10 cm trough space per hen).

### 2.3. Egg Sample

A sample of eggs was collected from each laying house at approximately 07:00–08:00 h, prior to commercial egg collection commencing. Four eggs were collected from one side of each aisle (one from each end and two from the middle), prior to commercial egg collection starting. Each egg was separated and the albumen frozen and stored at −20 °C until it could be analysed for corticosterone content by a third party using competitive protein binding radioimmunoassay [13]. The average value of albumen corticosterone content (ng/g) of all eggs in each sample was calculated to create a single value for each laying house (Corticosterone).

### 2.4. Behavioural Testing of Laying Hens

All behavioural tests were conducted in the morning following egg sample collection. The time taken to conduct the tests varied with the size of the flock but was approximately 2–3 h.

#### 2.4.1. Approaching Human Test

The Approaching Human Test (AHT) was adapted from a test used by Hemsworth and Barnett (1993) [10], and assessed the avoidance response of caged laying hens to an unfamiliar human approaching their cage in a standard manner. Every tenth cage on the second tier on the right-hand side of each aisle was designated as a focal cage. The second tier was approximately 1 m from the ground, although the exact height varied between farms. The same person (L.E.E.) was used as the human stimulus in all behavioural tests. This researcher was always dressed in a standard manner (blue overalls and a white dustmask), and her hands remained in her pockets. This attire was visually distinct from the clothing worn by stockpeople, who did not wear dustmasks. An assistant was dressed in a similar manner to the researcher (overalls), but held a stopwatch and clipboard and did not wear a white dustmask. The assistant remained at a distance of at least 2 m from the focal cage but was still visible to the hens being tested.

**Figure 1:**
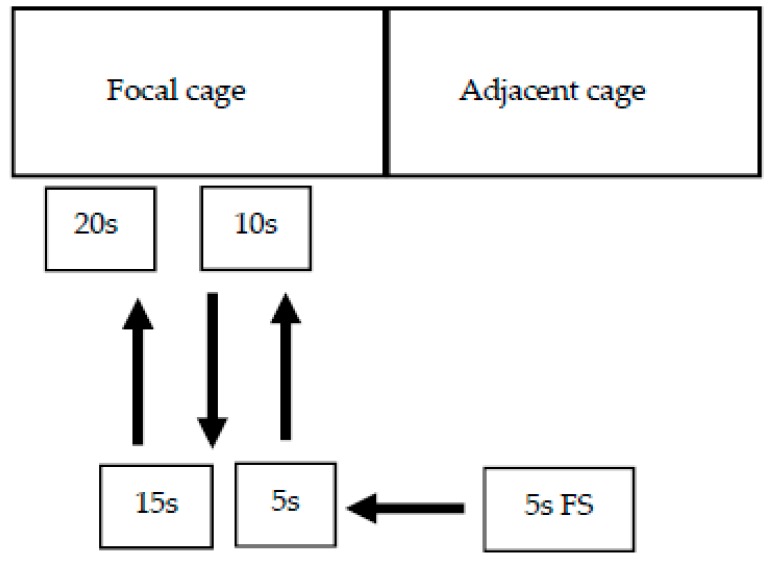
Movement of the researcher during the four stages of the Approaching Human Test. 5s FS = 5-second familiarization stage prior to the test commencing.

Each focal cage was tested once, and the testing occurred sequentially along each aisle. Each test consisted of five stages, and each stage was 5 s in duration. The first stage was a familiarisation stage, and allowed the hens in the focal cage to make visual contact with the researcher prior to the test commencing, avoiding a startle response. During this familiarisation stage the researcher stood on the opposite side of the aisle (approximately 1 m) to the cage that was adjacent to the focal cage to be tested. The researcher remained in this position for a period of 5 s, after which she stepped sideways so that she was directly in front of the focal cage and the Approaching Human Test began. After 5 s had elapsed, the researcher stepped toward the cage so that her torso was in contact with the feed trough at the front of the cage (approximate 20 cm from the cage front). The researcher waited in this position, observing the birds, for 5 s before stepping back to the opposite side of the aisle. The researcher stood in the original starting position for 5 s, after which she again stepped forward to the front of the focal cage and waited in this position for a further 5 s. Thus, the entire test for each cage took 20 s, and consisted of four movements made at 5-s intervals, depicted in Figure 1.

Whilst the researcher was standing stationary during each 5-s interval she made the following observations for each focal cage, and verbally relayed these to the assistant standing 2 m away:

- The maximum proportion of hens that simultaneously placed their beaks in the front 5 cm of the cage at any point during the 5-s period (variable labelled ‘Maximum Heads’);
- The proportion of hens with their beaks in the front 5 cm of the cage at the end of the 5-s period (variable labelled ‘Point Count’);
- The maximum proportion of hens that simultaneously placed their heads out of the cage at any point during the 5-s period (variable labelled ‘Heads Out’).

This resulted in 12 behavioural measurements for each focal cage (three variables measured four times each). A principal components analysis was used to reduce the number of variables. Two components of behaviour accounted for 82% of the variation in avoidance response. The first component was composed of the variables ‘Maximum Heads’ at 5, 10, 15 and 20 s, the ‘Point Count’ at 5, 10, 15 and 20 s, and ‘Heads Out’ at 5 s. These nine variables were combined into a single score that represented the first behavioural component and was labelled the ‘Forward Score’ (Cronbach’s alpha = 0.94). The second component was composed of the variable ‘Heads Out’ at 10, 15 and 20 s. These variables were combined into a single score that represented the second behavioural component, labelled the ‘Heads Out Score’ (Cronbach’s alpha = 0.86). A high value for either score indicates a greater proportion of birds at the cage front (Forward Score) or with their heads out (Heads Out Score) during the Approaching Human Test and thus indicating less avoidance of the researcher.

#### 2.4.2. Stroll Test

The Stroll Test was adapted from a similar test used by Cransberg et al. (2000) [14], and assessed the avoidance response of caged laying hens to an unfamiliar human walking along the aisles. The procedure involved the researcher walking through the laying house at a standard speed of one step/second and filming the response of the birds in the second tier with a hand-held video camera. The test was administered in one movement through the laying house, filming the same side of each aisle that the Approaching Human Test had been administered. The angle of the camera was held such that the distance to the first bird with its head out of the cage was able to be recorded, with a field of view of at least four cages ahead of the researcher.

During subsequent video analysis the footage was paused at 5-s intervals and the withdrawal distance was measured. The withdrawal distance was classified as the distance (cm) between the experimenter and the first bird with its head out of the cage, calculated by counting the number of cages between the two and multiplying by the cage width in cm (Withdrawal Distance). This variable measured the distance at which the hens would let the researcher approach before withdrawing their head into the cage. A greater ‘Withdrawal Distance’ indicates greater avoidance of the researcher.

The second variable measured was the number of birds with their heads extended through the front of the four cages directly ahead of the researcher. Only four cages were counted due to low visibility beyond this distance. The total number of birds with their heads out of the four cages was summed, converted to a proportion of the total number of birds in those four cages, and divided by the total length of the four cages in m. This allowed the proportion of birds with their heads out per m to be calculated, creating the variable ‘Prop Heads Out/m’. A greater value for the ‘Prop Heads Out/m’ indicates less avoidance of the researcher. This standardised unit of measurement was calculated to account for the between-farm differences in cage widths and stocking densities.

### 2.5. Stockperson Behaviour Observations

The behaviour of all stockpeople that entered the laying house was observed over a 2-day period. These observations are referred to as ‘All SP’ and represent the total human behaviours that the hens were exposed to. An ethogram of all stockperson behaviours recorded and how they were categorised is presented in Table 2. A subset of these observations are the behaviours performed by the focal stockperson only, referred to as ‘Main SP’. The focal stockperson was the main person that was responsible for the maintenance and husbandry within each house, and spent more time in the laying house than other farm staff. The focal stockperson behaviours were used to assess the relationships between stockperson attitudes and behaviours.

The researcher (L.E.E.) followed the stockperson at a short distance (10–15 m) and verbally recorded observations on a small handheld tape recorder in real time. The frequency of each stockperson behaviour was recorded when analysing the cassette tapes at a later date, and a criterion of 5-s was used to record the frequency of behaviours that were performed for >5-s. For example, a behaviour that occurred for 8-s would be classified as occurring twice. The stockpeople were informed that their activities in the house were being observed, and were instructed to continue their work as if the researcher was not present. Due to the large number of different stockperson behaviours observed, all behaviours were grouped on the basis of their type of interaction with the birds, resulting in seven categories of stockperson behaviour (Visual, Noise, Approach, Contact, Entry, Handle, Near Cage).

In addition, the researcher also recorded the amount of time (seconds) that each stockperson spent in the aisles (Time in Aisle); at either the start or end of the laying house (Time SOH); in total for both the aisles and the start of the laying house (Total Time); at the end of each aisle without walking the entire length of the aisle (such as when maintaining equipment, Time Ends Aisles); and the time spent standing stationary in the aisles (Stationary). Speed of movement was calculated by dividing the time spent walking along an aisle by the length of the aisle (Av SOM, Min SOM, Max SOM, m/seconds).

The total numbers of occurrences of behaviours in each category were summed for each laying house over the 2-day observation period, divided by two to calculate the average number of each behaviour per day, and then divided by the total number of cages in the laying house to provide a standard measure of stockperson behaviour per cage. This conversion allowed the behavioural observations to be converted into a standard unit (average behaviours/cage/day) for comparison between laying houses of different sizes. The duration of time that individual stockpeople spent in each area of the laying house (such as the aisles or ends of the laying house) was also converted into the units ‘seconds in each area per cage’. Prior to inclusion in statistical analysis, several of these measurements were transformed to reduce skewness in the measurement. The transformations used for each variable are included in the results tables.

### 2.6. Stockperson Attitude Assessment

Stockperson attitudes were assessed using a written questionnaire. The questionnaire was administered to the focal stockperson working in each house at the end of the 2-day behaviour observation period. Complete questionnaires were obtained for 14 stockpeople.

The questionnaire consisted of three sections: the first section contained statements about working with laying hens and assessed stockperson beliefs about the behaviours that they performed when working in the laying houses (behavioural beliefs); the second section contained statements about the characteristics of laying hens and assessed general beliefs about laying hens; and the third section contained statements about interacting with laying hens and assessed behavioural beliefs about interacting with laying hens. Stockpeople were asked to indicate the extent to which they agreed or disagreed with these statements using a five-point Likert scale, ranging from ‘Strongly agree’ to ‘Strongly disagree’ with ‘Neither agree or disagree’ as the middle option. Each stockperson was given a score from 1–5 for each answer, with ‘Strongly disagree’ receiving a score of one and ‘Strongly agree’ receiving a score of five.

Initial questionnaire items were developed from focus group discussions with stockpeople working in the egg industry. The large number of initial questionnaire items could not be reduced using a principal components analysis due to the small sample size, and so the questionnaire items were manually sorted into subscales with similar content using item-total correlations to exclude non-correlated items in each subscale. This resulted in 10 subscales, and the scores for the items in each subscale were summed to create a single value for each subscale for each stockperson. All of the items in a subscale were significantly correlated with the subscale total, and all subscales had a Cronbach’s alpha score above 0.7 indicating that each total was a reliable measure of each subscale. Each subscale is described below.

Statements relating to the stockperson’s general beliefs about the characteristics of laying hens were divided into positive and negative statements about laying hens. The positive statements formed the first attitude subscale ‘Pos Gen Atts’ (Cronbach’s alpha = 0.83), which consisted of nine statements such as ‘Laying hens are entertaining to watch’ and ‘Laying hens are intelligent animals’, and stockpeople who agreed with these statements were considered to have a positive general attitude toward laying hens. Conversely, negative statements about the characteristics of laying hens were grouped to form the second attitude subscale ‘Neg Gen Atts’ (Cronbach’s alpha = 0.81) and consisted of 13 statements such as ‘Laying hens are dirty animals’ and ‘Laying hens have an ugly appearance’. Stockpeople who agreed with these statements were considered to have a negative general attitude toward laying hens.

Statements relating to the stockperson’s behavioural beliefs about interacting with laying hens were also grouped into subscales. The third subscale was labelled ‘Insensitivity of SP’ (Cronbach’s alpha = 0.75), and consisted of 13 statements such as ‘Yelling at the birds quietens them down’ and ‘Laying hens aren’t affected by the way they are treated’. Stockpeople who agreed with these statements were considered to be unaware of or unconcerned with the effects that their behaviour had on the hens. Conversely, the fourth subscale labelled ‘Sensitivity of SP’ (Cronbach’s alpha = 0.74) consisted of five statements such as ‘I should act carefully around laying hens so as not to scare them’ and ‘I notice differences in the way laying hens respond to me’. Stockpeople who agreed with these statements were considered to be aware of the impact of their behaviour on the hens. The fifth attitude subscale was labelled ‘Unpleasantness of Job’ (Cronbach’s alpha = 0.87) and consisted of 13 statements such as ‘Laying hens are frustrating to work with’ and ‘I find the laying house too noisy’. Stockpeople who agreed with these statements were considered to find their work unpleasant, or dislike certain aspects of their job. The sixth attitude subscale was labelled ‘SP Enjoys Job’ (Cronbach’s alpha = 0.79), and consisted of statements such as ‘I don’t mind the dust in the laying house’ and ‘I am happy with the amount of walking that I have to do in the laying house’. Stockpeople who agreed with these statements were considered to enjoy their work, or find some aspects of their work pleasant. The seventh attitude subscale was labelled ‘No Job Control’ (Cronbach’s alpha = 0.73) and consisted of six statements such as ‘I don’t have much control over what I do in the laying house’ and ‘I have no input into how my job is done’. Stockpeople who agreed with these statements were considered to have little perceived control over the work that they do, and felt unvalued. The eighth attitude subscale was labelled ‘Job Control’ (Cronbach’s alpha = 0.70) and consisted of two statements, ‘Management listens to my suggestions’ and ‘I am an important member of a team’. Stockpeople who agreed with these statements were considered to feel valued, and able to contribute to how well his or her job was done. The ninth attitude subscale was labelled ‘Diligence’ (Cronbach’s alpha = 0.76) and consisted of eight statements such as ‘’I try to make the hens as comfortable as possible’ and ‘I am very thorough in my work’. Stockpeople who agreed with these statements were considered to value their work, and place emphasis on doing their work thoroughly and correctly.

The final subscale related to the empathic capabilities of the stockperson rather than their attitudes. However, for the sake of brevity, the empathy subscale will be included in the term ‘attitude subscales’ throughout this paper. Labelled ‘Empathy’ (Cronbach’s alpha = 0.81), this subscale consisted of four statements such as ‘It is kinder to handle the birds gently’ and ‘I feel bad if the hens go without food or water’. Stockpeople who agreed with these statements were considered capable of vicariously feeling a similar subjective state to that of the hens, and were concerned with the comfort of the hens.

### 2.7. Productivity Records

The production records were obtained from the farm managers at the conclusion of the study. The values for peak hen day production (PHDP), age at peak hen day production (Age at PHDP) and rate of lay in the week of testing (hen day production, HDP) were determined for each flock. The number of production weeks within 5% of peak egg production were calculated as a measure of persistency of lay [15]. Due to the age-related variation in hen productivity, the productivity variables for each flock were standardised by comparing them to the appropriate production standard [16,17] for each age (week) for each strain of hen, rather than between flocks of different ages. The standard value for each productivity variable was subtracted from the actual value for each variable. Thus, a negative value for these productivity measures meant that the flock had a lower productivity than expected at that age for that strain, and a positive value meant that the flock was producing better than expected at that age. The cumulative mortality rate on the week of testing was also determined for each flock and the production standard value subtracted to create the standardised variable ‘Mortality’.

A list of all the variables collected in this study along with their descriptive statistics are presented in Table 3.

### 2.8. Statistical Analysis

The general approach was to select an outcome variate related to productivity (5% peak egg production duration) that had a likelihood of being directly or indirectly affected by stockperson behaviour and hen fear and stress. This variable was chosen based on the results of preliminary analyses (unpresented) that revealed few likely relationships between other production variables and hen behaviour variables. For example, an analysis relating peak hen day production (PHDP) to albumen corticosterone, laying house parameters, hen avoidance behaviours, stockperson behaviours and stockperson attitudes was attempted. The parsimonious model for the logarithm of PHDP only included laying house terms. In particular, PHDP increased as the size (in terms of flock size) of the laying house increased and the light level at the cage front (Lux) increased. These responses do not appear to be directly related to a stockperson-animal relationship, and thus the detail of this analysis is not reported in this paper.

On a between laying house basis, a parsimonious statistical model was developed relating 5% peak egg production duration to albumen corticosterone, laying house parameters (e.g., bird strain, number of birds, cage design etc.), hen avoidance behaviours, stockperson behaviours and stockperson attitudes. There were only three predictors in the parsimonious model (see results for detail), namely:

- A predictor associated with cage design, namely cage width;
- A predictor often associated with animal fear, namely average withdrawal distance during the Stroll Test (AvWD);
- A predictor related to stockperson behaviour, namely the square root of the frequency of noise behaviours of all stockpeople (AllSPNoise).

To understand the HAR further, the average withdrawal distance of each laying house was related to the physical features of the laying house, all stockperson behaviour measurements, main stockperson behaviour measurements and main stockperson attitude measurements. As the results of the AHT were not predictive of the number of weeks that the hens spent within 5% of peak egg production, these results were not analysed further. If a measurement is a predictor of the average withdrawal distance, without being a predictor of the outcome variate in the parsimonious model (i.e., no effect on the outcome variate after adjusting for the effect of average withdrawal distance), then the measurement is a candidate for mediating or affecting productivity via the mechanism of affecting the average withdrawal distance.

To understand the factors that influence the amount of noise made by the main stockperson, the square root of the frequency of noise behaviours of the main stockperson (square root of MainSPNoise) for each laying house was related to physical features of the laying house, all stockperson behaviour measurements other than noise behaviour, main stockperson behaviour measurements other than noise behaviour and main stockperson attitude measurements. The main stockperson noise behaviour, rather than all stockperson noise behaviour, was used as the dependent variate because attitude measurements were only available for the main stockperson. Stockperson behaviour measurements, other than noise behaviour, were included as predictor variates for MainSPNoise because, while noise behaviour is a stockperson behaviour, it is partly determined by management decisions like the cleaning routines in the laying house.

Finally, since fear-related stress is considered to be an important component of the HAR, the logarithm of albumen corticosterone was related to physical features of the laying house, hen avoidance behaviours, stockperson behaviours and stockperson attitudes. Cage width was not considered further because its effect on the outcome variates was considered likely to be caused by farm management decisions, and thus not influenced by the stockperson.

Using residual maximum likelihood (REML) mixed model analysis, a parsimonious model was developed to relate 5% peak egg production duration, of each laying house, to fixed effects for the logarithm of albumen corticosterone, features of the laying house, avoidance behaviour test results, all stockperson behaviour measurements, main stockperson behaviour measurements and main stockperson attitude measurements and random effects associated with farm identity. Prior to examination of some fixed effects that had skewed distributions, those effects were transformed to reduce the skewness of the distribution. Fixed effects were included or excluded from the models using Wald F tests, and random effects were included or excluded using χ^2^ change in deviance tests. Random effects for farm are likely to be necessary because animal productivity is likely to be affected by unmeasured factors outside the HAR paradigm (such as nutrition and genetics), and these factors are likely to be determined on a farm basis. This is likely to induce correlation between different laying houses on the same farm, which in turn induces correlation in the errors of the model. Confidence intervals for predictors in the parsimonious model were calculated on the logarithmically transformed scale, using the asymptotic normal distribution, before back-transforming to the original albumen concentration scale.

For each of the logarithm of albumen corticosterone, average withdrawal distance and the square root of MainSPNoise, a parsimonious general linear model was developed to relate the measurement to the sets of predictor variates in the paragraphs above. Terms were included and excluded from the models using standard F tests for linear models. To examine whether systematic variation between farm needed to be included in the models, for each of the logarithm of albumen corticosterone, average withdrawal distance and the square root of MainSPNoise, an attempt was made to fit a residual maximum likelihood (REML) mixed model that included the parsimonious model terms plus an extra random term for farm identity. However, in each case the best estimate of the variance for the farm identity effect was negative. Since it is implausible that the true variance is negative, although a negative estimate often occurs when the variance is zero, a random effect for farm was not included in these models. Confidence intervals for predictors in the model for MainSPNoise were calculated on the square root scale, using the t distribution, before squaring to obtain a confidence interval on the original MainSPNoise scale.

The unit of all analysis was a single laying house. Statistical analyses were carried out using the general linear model component, and the REML analysis of mixed models component, in GenStat 17 [18].

## 3. Results

The sheds were representative of the commercial caged egg industry in south east Australia (Table 1). There was almost a 100-fold difference in shed size, with sheds varying from 1300 birds to 116,000 birds

### 3.1. The Number of Weeks within 5% of Peak Egg Production

The most parsimonious model for 5% peak egg production duration included a random term for farm (χ^2^ change in deviance = 12.69 on 1 degrees of freedom; *p* = 0.00089) and additive fixed effect terms for cage width, a linear response to average withdrawal distance in the Stroll Test and the noise behaviour of all stockpeople (Table 4). The cage width effect could be described as having two groupings, namely narrower cages (≤50 cm) and wider cages (>60 cm), with no further effect of cage width (*p* = 0.32 for additional linear response to cage width, Table 4). Chi-square change in deviance tests were also carried out, using random coefficient regression models and variance component models, to examine whether any of the fixed effects varied randomly with farm but, in all cases, either the models were not statistically significant (*p* > 0.1) or did not numerically converge.

**Figure 2:**
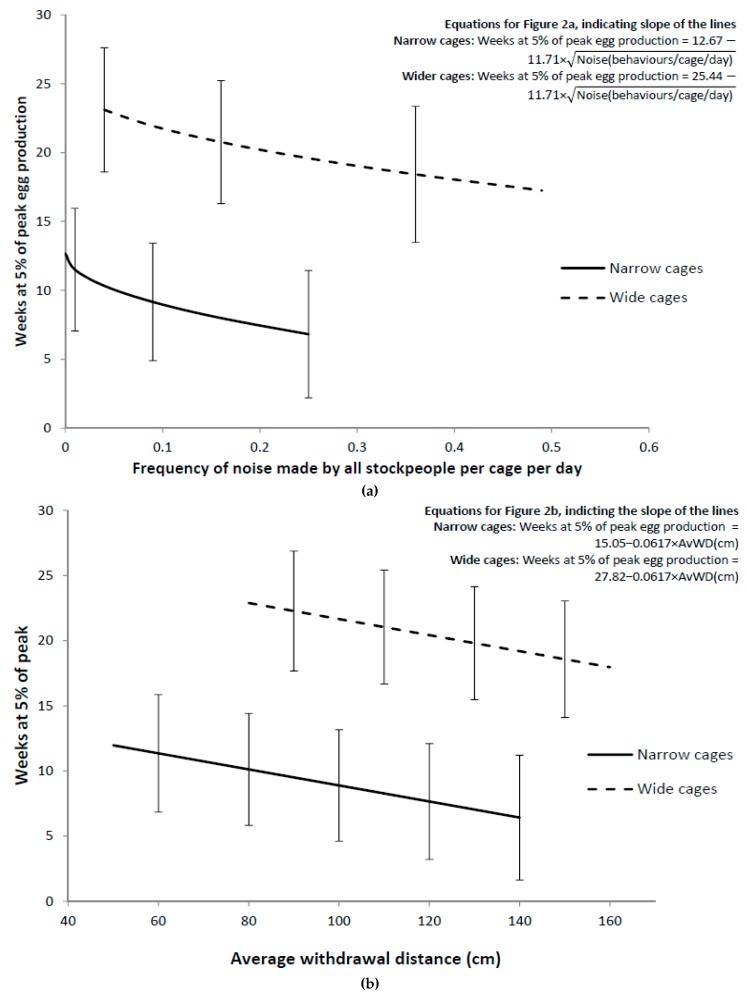
(**a**) Relationship between weeks at 5% of peak egg production and all stockperson noise, after adjusting for average withdrawal distance (AvWD), for the two cage width groupings. The noise responses, for each grouping, are presented at the between shed mean of average withdrawal distance, for sheds in that grouping. (**b**) Relationship between weeks at 5% of peak egg production and AvWD, after adjusting for the square root of all stockperson noise, for the two cage width groupings. The AvWD responses, for each grouping, are presented at the between shed mean for all stockperson noise, for sheds in that grouping. Error bars are 95% confidence intervals using the asymptotic normal approximation.

The largest effect was the effect of cage width grouping, with the wider cages (>60 cm) having at least a 10 week greater 5% peak egg production duration than narrow cages (≤50 cm), for laying houses with the same average withdrawal distance in the Stroll Test and noise behaviour made by all stockpeople (Figure 2). Despite the wide confidence limits in the predicted means in Figure 2a,b, the marginal slope for 5% peak egg production duration (weeks) on the square root of noise made by all stockpeople (behaviours/cage/day) and the marginal slope of 5% peak egg production duration slope per day on average withdrawal distance (cm) are greater than three (slope = −11.7 (SE = 3.29) for square root of noise frequency; slope = −0.062 (SE = 0.0192) for average withdrawal distance), and thus there is reasonably good precision for the estimation of these slopes. For laying houses with the same width cages, the number of weeks spent within 5% of the peak in egg production decreased by about 5 weeks from the laying houses with the least noise made by all stockpeople (AllSPNoise) to the laying houses with the most noise made by all stockpeople. There was also a 5-week difference between the laying houses with the smallest average withdrawal distance to the laying houses with the greatest average withdrawal distance.

### 3.2. Albumen Corticosterone Concentration

**Figure 3:**
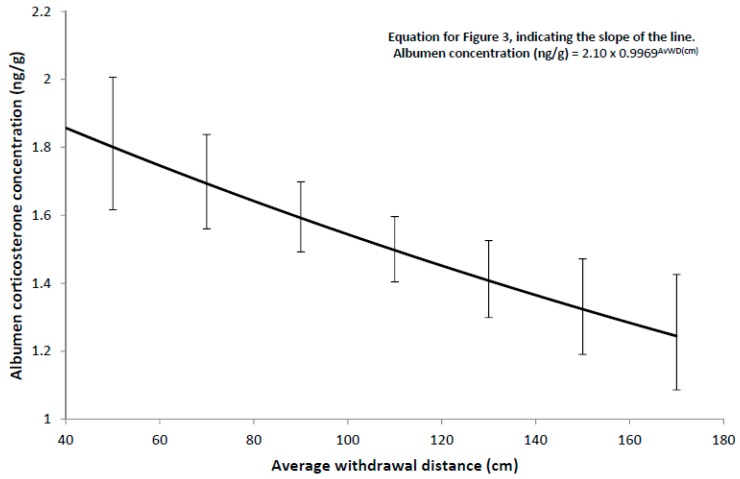
Relationship between albumen corticosterone concentration and the average withdrawal distance in the Stroll Test. Error bars represent 95% confidence intervals.

The most parsimonious model for the logarithm of albumen corticosterone concentration only included a negative linear response to the average withdrawal distance in the Stroll Test (i.e., a single linear regression on average withdrawal distance, Table 5). The average withdrawal distance of hens was strongly related, in a negative direction, to albumen corticosterone concentration (Figure 3).

### 3.3. Average Withdrawal Distance during the Stroll Test

**Figure 4:**
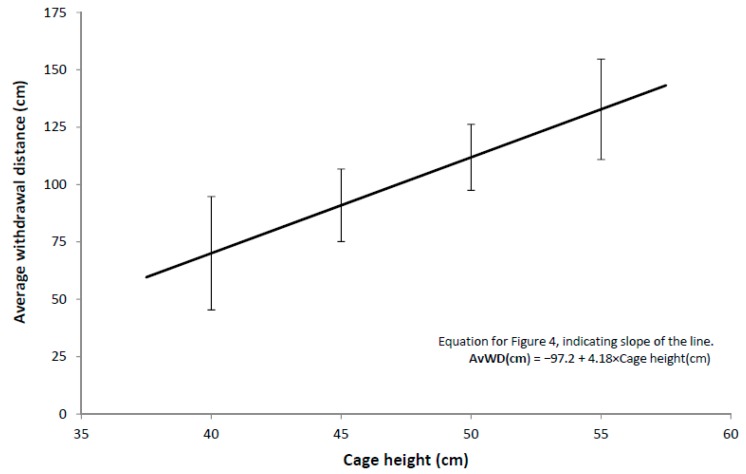
Relationship between average withdrawal distance in the Stroll Test and cage height. Error bars represent 95% confidence intervals.

The average withdrawal distance during the Stroll Test was the only hen behaviour variable that was associated with the 5% peak egg production duration and the albumen corticosterone concentration. For this reason, the results of the AHT are not presented. The most parsimonious model for the average withdrawal distance only included a positive linear response to cage height (i.e., a single linear regression on cage height, Table 6, Figure 4). Two stockperson behaviours (proportion of time at the end of the aisles, both for all stockpeople and for the main stockperson) had *p* values between 0.05 and 0.03 when added to the parsimonious model, but two out of 32 stockperson behaviours being statistically significant at the 5% level were judged to be insufficient to be reliable evidence of an effect, and thus these behaviours were not included in the parsimonious model. After adjusting for cage height, there was also a slight negative relationship between the average withdrawal distance and the insensitivity score for the main stockperson (*p* = 0.05, Table 6).

### 3.4. Noise Made by the Main Stockperson

The most parsimonious model for the frequency of noise (square root transformed) made by the main stockperson included additive linear responses to the square root of all stockperson hand entries per cage, main stockperson positive general attitude score and main stockperson insensitivity score (i.e., a multiple linear regression for square root of all stockperson entries per cage, main stockperson positive general attitude score and main stockperson insensitivity attitude score, Table 7).

**Figure 5:**
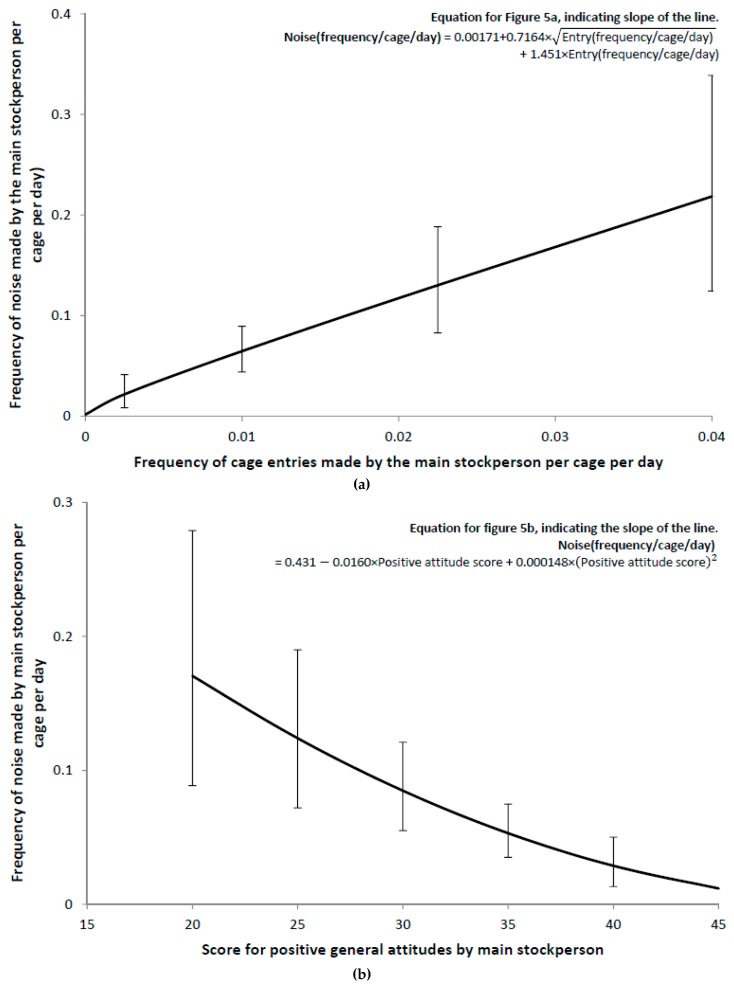

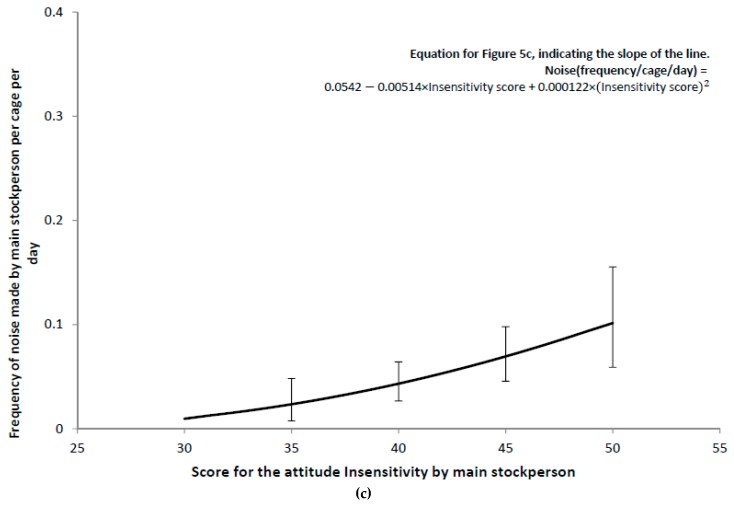
(**a**) Relationship between main stockperson noise and all stockperson entries per cage, after adjusting for stockperson positive attitude score and stockperson insensitivity attitude score. (**b**) Relationship between main stockperson noise and main stockperson positive attitude score, after adjusting for square root of all stockperson entries per cage and stockperson insensitivity attitude score. (**c**) Relationship between main stockperson noise and main stockperson insensitivity attitude score, after adjusting for square root of all stockperson entries per cage and stockperson positive attitude score. Error bars represent 95% confidence intervals.

The amount of noise made by the main stockperson was greater when the number of cage entries made by all stockpeople in the shed was greater, and this was the strongest relationship with noise (Figure 5a). The main stockpeople also made more noise when they had a low score for positive general attitudes and a high score for the insensitivity attitude (Figure 5b,c).

## 4. Discussion

The human-animal relationship model predicts that there is a relationship between the attitudes of stockpeople and their behaviour toward animals, which in turn influences the welfare of farm animals by mediating their fear of humans and the associated stress response, with subsequent reductions in productivity. The results of the current study will be discussed in the reverse order of this model, starting with the productivity results, so that only the relevant variables linking each section of the model are presented. It needs to be recognised that while there were significant relationships found between several variables in this study, these relationships were observational and thus cannot be interpreted as causal until they are tested using controlled experiments.

The productivity of the hens (the number of weeks producing within 5% of the peak in egg production) was most closely related to whether cage width was less than 50 cm or greater than 60 cm (there were no cages between these two widths). Cages that were wider than 60 cm showed an additional 10 weeks of sustained high egg production compared to cages that were 50 cm wide or less. While it is tempting to attribute this effect to the wider feed trough decreasing competition and increasing the availability of feed to the hens [19], this explanation does not take into account the group size within the cage, which will affect the amount of feeder space available to each hen. In addition, the duration of peak egg production did not vary within each of the cage width categories, suggesting the effect is not linearly dependent on cage width. Thus, the difference could partly be due to other aspects of the design of cages that happen to be associated with width but were not included in the analyses (cage depth, cage height and group size were included). This may include variables such as the amount of light or air movement at the cage front. The conclusion from this part of the results is that cage design may be very important to productivity, rather than cage width *per se* being the important factor.

Hens with similar cages were able to maintain a higher production curve (duration within 5% of peak egg production) for longer when they allowed the researcher to approach more closely before withdrawing, and when they were exposed to quieter stockpeople. The model estimates that, for hens from laying sheds with a similar cage width grouping, an additional 5 weeks of high production can be obtained for flocks that showed the lowest withdrawal distance compared with those with the greatest withdrawal distance. Additionally, for hens from laying sheds with similar cage design, an additional 5 weeks of high production can be obtained for flocks that were exposed to the least amount of stockperson noise compared to those exposed to the greatest amount of stockperson noise. This is a promising result, as it indicates that both fear of humans and stockperson behaviour may influence egg production, and is consistent with a fear-related stress response [1,3,20]. For example, Barnett et al. (1992) [12] found that commercial caged laying hens could maintain egg production within 5% of their peak for longer when they showed less avoidance in a human approach test. In addition, O’Connor et al. (2011) [21] found that laying hens exposed to chronic noise at 80 dB produced less eggs, with no appreciable increase in physiological stress (based on plasma corticosterone concentration and heterophil:lymphocyte ratios), similar to the current study. Much of the noise made in the laying houses was created by loud cleaning machinery, such as leaf blowers and air hoses that were used to blow the dust off horizontal surfaces. This machinery could be used for long periods, and substantially increased the amount of airborne dust within the laying house during cleaning.

Negative relationships between hen avoidance behaviour and productivity are usually assumed to be due to a fear-related stress response. However, the result that the parsimonious model for 5% peak egg production duration included a term for average withdrawal distance, but no term for albumen corticosterone concentration, indicates that this mediation is not occurring.

In addition, the baseline stress level of each flock, as indicated by average albumen corticosterone concentration, was lower for flocks that showed a greater withdrawal distance toward an approaching unfamiliar human. This is the opposite to what would be expected with a fear-related explanation of the relationship between hen avoidance behaviour and productivity. While these two results are contrary to the previously established positive relationship between fear of humans and stress physiology in laying hens [22,23,24], a recent study with pullets has found the same relationship. Sprafke et al. (2018) [25] reported that the number of pullets in a cage-free flock that could be physically touched by the researcher was greater when the pullets had higher basal corticosterone concentrations, as indicated by the concentration of corticosterone metabolites in their droppings. No explanation for this result was provided by the authors. Albumen corticosterone concentration has been successfully correlated with plasma corticosterone concentration [13,26], (although see Engel et al. (2011) [27] for an opposing view), and while plasma corticosterone was not measured in the current study, albumen corticosterone concentration was negatively correlated with space allowance (r = −0.46, *p* = 0.048) as expected [28]. This indicates that the albumen corticosterone measurements are accurate, and a definitive explanation for this apparently anomalous result is not available.

The relationship between withdrawal distance and cage height may be due to the greater mobility of these birds to move backward within the cage when approached, as conventional cages have sloped floors and the available space at the back of the cage may be limited by cage height. Alternatively, due to the additive effects of cage height, hens in taller cages are likely to be closer to the researcher’s face during the behavioural testing. Hens will show longer tonic immobility [29] and greater avoidance of humans [30] when the researcher’s eyes are visible during testing. It is possible that closer proximity to the researcher’s face may have provoked a greater avoidance response. As the exact height of the cages from the ground or the roof height at the back of the cage was not measured in this study, these effects could not be directly examined.

A major prediction of the human-animal relationship model is that the avoidance response of the laying hens would be related to the behaviour and attitudes of the stockperson. This was not the case in the current study, in which the parsimonious model for average withdrawal distance included no terms for stockperson behaviour or attitudes. If attitudes and behaviour are related to fear of humans in hens then, at least after adjusting for the effect of cage height, a relationship between average withdrawal distance and stockperson behaviour would be expected. The result that no such relationships were observed indicates that stockperson behaviours did not substantially affect fear of humans in the hens. This is in contrast to recent research on the human-animal relationship by Waiblinger et al. (2018) [8], who found relationships between stockperson attitudes, stockperson behaviour and hen avoidance behaviour on free-range egg farms.

Despite this, after adjusting for terms that might not be associated with the human-animal relationship, a shorter duration spent within 5% of peak egg production was associated with more stockperson noise, and the amount of noise produced by an individual stockperson was associated with the attitudes of that stockperson and the number of cage entries made by all stockpeople in the laying house. Stockpeople made more noise when they had negative attitudes towards the hens, indicated by scoring a low value for Positive General Attitudes, or a high value for Insensitivity. The amount of noise made by stockpeople was also greater when all people working in the laying house put their hands in the cages more often (cage entries). There is no obvious explanation for this relationship, but it may be related to farm management decisions or the design of the laying house influencing cleaning and inspection procedures.

These attitude-behaviour relationships are in the expected direction [1] and may represent the degree of volitional control that the stockperson has over their noise-related behaviours. For example, a stockperson who is unaware or insensitive to the impact of their behaviour on the hens may not moderate their noise levels as much as a stockperson who is aware of their impact. Similarly, a stockperson with few positive general attitudes toward hens may be aware of the impact that noise has on the hens but may have little motivation to reduce that impact by moderating noise production. The observed relationships between stockperson attitudes and noise behaviour are consistent with previous research on the human-animal relationship [1].

## 5. Conclusions

In conclusion, the major prediction of the HAR that the behavioural and physiological responses of hens to an approaching human would be related to the attitudes and behaviours of stock people did not hold, on a between laying house basis, for commercial caged hens in Australia. There was evidence of a relationship between stockperson attitudes and noise behaviour, and a high frequency of noise was associated with a reduction in one measure of egg production. If noise was affecting hen productivity it appears not to be mediated through its effects on fear of humans in hens. In addition, flocks that showed the least avoidance of the researcher had the greatest concentration of corticosterone in their egg albumen. Clearly further research is required to more thoroughly understand this human-animal relationship and its implications for hen welfare and productivity, on Australian cage-egg farms. The imperative for this is the findings by Waiblinger et al. (2018) of relationships between stockperson attitudes and behaviour and hen avoidance behaviour and productivity on free-range egg farms.

## Figures and Tables

**Table 1:** A description of each of the laying houses used in the study, highlighting variation in design and genetics.

Farm	Laying House within Farm	Flock Size (in ‘000s)	No. of Stock People per House	No. of Tiers	Av. No. of Birds per Cage	Aisle Length (m)	Age of Birds (weeks)	Strain of Birds	No. of Focal Cages per House
1	a	14	5	3	2.9	86	50	IB	77
2	a	12.3	4	3	3.0	84	57	HB	90
3	a	24	6	4	4.7	74	57	IB	61
4	a	2.5	2	1	2.9	30	48	IB	52
5	a	1.3	2	1	3.0	39	48	HB	34
6	a	22	6	4	3.7	85	55	IB	87
6	b	68.3	9	8	6.2	83	52	IB	74
6	c	22	4	4	3.9	84	90 ^†^	IB	87
7	a	22	4	4	5.8	54	36	IH	58
8	a	29.4	1	5	8.9	76	48	HB	88
8	b	21.9	4	4	5.6	74	51	HB	62
9	a	108.1	5	6 *	6.2	129	69	IB	159
9	b	122	6	6 *	4.7	129	66	IB	107
9	c	116.4	5	6 *	6.8	129	62	IB	80
9	d	116.4	4	6 *	6.5	129	60	IB	80
10	a	67.5	5	6 *	5.3	93	65	IB	91
10	b	67.5	10	6 *	6.4	93	63	HB	90
10	c	67.5	5	6 *	7.0	93	42	HB	91
10	d	67.5	8	6 *	6.8	93	43	HB	91

* Denotes laying houses with six tiers of cages that had a wire mesh mezzanine floor installed between tiers 3 and 4. Stockpeople walked on the ground floor to monitor tiers 1–3, and on the mezzanine floor to monitor tiers 4–6. Husbandry observations were only made on the ground floor as this was the location of the focal birds. ^†^ Denotes a 90 wk old flock that had been moulted to extend the productive life of the hens. The number of birds in each focal cage varied due to mortalities. IB = ISA Brown, HB = Hyline Brown, IH = Ingham Hisex.

**Table 2:** An ethogram of the specific stockperson behaviours that were recorded, and how they were grouped into each behavioural category.

General Behaviour Category	Definition of Behaviour	Specific Stockperson Behaviours
Visual	Any behaviour that was performed in front of the cages, but did not involve noise or approaching within 20 cm of the cage.	Manipulate clothing Manipulate object Pause Push object Carry object Kick object Sweep Crouch Turn around Bend down Drop object
Noise	Any behaviour that generated noise.	Noise (e.g., knock broom on cage) Yell Loud noise (e.g., leaf blower)
Approach	Any behaviour that involved a body part or an object held by the stockperson to approach within 20 cm of the cage without contacting the cage.	Close visual inspection of cage Throw object onto manure belt Hand approaches cage Torch use Face over feeder Object approaches cage Object over cage Object under cage
Contact	Any behaviour that involved a body part or object held by the stockperson to contact the cage, feed trough, egg belt or rail.	Contact feeder Lean on feeder Manipulate egg Object on egg belt Object in feeder Hand in feeder Hand under feeder Hand on rail Stand on feeder Stand on rail Stand on cage Hand on cage Manipulate cage Bang cage Kick egg belt Kick feeder Contact cage Contact feeder with object Contact cage with object
Entry	Any behaviour that involved inserting a hand or an object into the cage.	Insert object into cage Insert one hand into cage Insert both hands into cage
Handle	Any behaviour that involved touching or handling the hens.	Touch bird Handle bird
Near Cage		A composite variable made by summing the behavioural categories ‘Approach’, ‘Contact’, ‘Entry’, ‘Handle’

**Table 3:** Descriptive statistics of laying houses, for all variables included in this study.

Variables Measured	N	Mean	SD	Minimum	Maximum
Hen avoidance behaviour					
Forward Score	19	0.52	0.66	−0.38	1.87
Heads Out Score	19	0.16	0.63	−0.34	2.22
Withdrawal distance (cm)	19	101	36	46	170
Proportion heads out/m	19	0.25	0.13	0.06	0.46
Baseline stress physiology					
Albumen corticosterone concentration (ng/g)	19	1.56	0.27	1.10	2.18
Laying house features					
No. of stockpeople in house	19	5	2	1	10
Age of birds (weeks)	19	56	12	36	90
Flock size	19	51,193	41,163	1320	121,968
Length of aisle (m)	19	87	28	30	129
Cage width (cm)	19	54	12	31	70
Av. no. of birds/cage	19	5.3	1.7	2.9	8.9
Space allowance (cm^2^/bird)	19	538	84	382	689
Cage height (cm)	16	48.2	5.6	39.0	55.0
Cage depth (cm)	18	51.4	6.9	38.0	59.0
Av. lux at cage front	19	107.2	317.6	0.03	1248.7
All stockperson (SP) behaviours					
All SP Visual/cage/day	19	0.36	0.41	0.01	1.65
All SP Noise/cage/day	19	0.15	0.17	0.00	0.51
All SP Approach/cage/day	19	0.11	0.06	0.00	0.22
All SP Contact/cage/day	19	0.22	0.34	0.02	1.21
All SP Entry/cage/day	19	0.01	0.01	0.00	0.04
All SP Handle/cage/day	19	0.005	0.01	0.00	0.04
All SP Near Cage/cage/day	19	0.34	0.34	0.07	1.25
All SP Time SOH/cage/day (second)	19	1.03	0.49	0.20	1.93
All SP Time in aisles/cage/day (second)	19	3.78	1.81	1.45	8.89
All SP Total time/cage/day (second)	19	3.82	1.99	0.85	8.82
All SP Time Ends Aisles/cage/day (second)	19	0.38	0.32	0.00	1.02
All SP Stationary/cage/day (second)	19	0.24	0.22	0.00	0.84
All SP Av SOM (m/second)	19	0.95	0.34	0.40	1.81
All SP Max SOM (m/second)	19	2.76	1.15	1.01	4.93
All SP Min SOM (m/second)	19	0.22	0.24	0.04	1.01
Main stockperson (SP) behaviours					
Main SP Visual/cage/day	14	0.17	0.15	0.01	0.46
Main SP Noise/cage/day	14	0.08	0.10	0.00	0.25
Main SP Approach/cage/day	14	0.06	0.04	0.01	0.16
Main SP Contact/cage/day	14	0.08	0.09	0.01	0.35
Main SP Entry/cage/day	14	0.004	0.004	0.00	0.02
Main SP Handle/cage/day	14	0.001	0.002	0.00	0.01
Main SP Near cage/cage/day	14	0.16	0.13	0.04	0.49
Main SP Time SOH/cage/day (second)	14	0.78	0.37	0.06	1.47
Main SP Time Ends Aisles/cage/day (second)	14	0.12	0.12	0.00	0.38
Main SP Stationary/cage/day (second)	14	0.11	0.09	0.00	0.33
Main SP Time In Aisles/cage/day (second)	14	1.27	0.70	0.21	2.49
Main SP Total time/cage/day (second)	14	1.81	0.87	0.51	3.46
Main SP Av SOM (m/second)	14	0.99	0.18	0.71	1.23
Main SP Min SOM (m/second)	14	0.21	0.27	0.04	1.01
Main SP Max SOM (m/second)	14	2.56	0.88	1.01	4.43
Main stockperson attitudes					
Empathy	14	14.2	3.3	6.0	17.0
Sensitivity of stockperson	14	35.7	5.7	25.0	43.0
Insensitivity of stockperson	14	41.7	6.3	32.0	51.0
Job control	14	7.6	1.8	2.0	10.0
No job control	14	16.5	4.1	9.0	25.0
Stockperson enjoys job	14	26.7	6.5	12.0	35.0
Diligence	14	33.6	6.1	20.0	41.0
Unpleasantness of job	14	31.7	9.8	17.0	51.0
Neg general attitudes	14	38.8	9.2	25.0	55.0
Pos general attitudes	14	35.3	7.0	20.0	44.0
Flock productivity					
Peak Hen Day Production (standardized %)	16	0.73	1.77	−2.60	3.88
Hen Day Production (standardized %)	15	3.37	1.78	0.80	7.12
Cumulative mortality (standardized %)	15	−1.47	2.09	−4.35	3.90
Age at Peak Hen Day Production (weeks)	16	9	7	1	21
Weeks at 5% of peak egg production (weeks)	16	16	7	1	27

All stockperson behavior units are frequencies/cage/day in the laying house except where followed by (second), in which case they are durations/cage/day. SP = stockperson, SOH = start of laying house, SOM = speed of movement, SD = standard deviation. Hen Day Production refers to the average % of hens laying an egg on each day over a 1-week period. All production data have been subtracted from the expected production values for flocks of the same strain and age to account for age differences in production between flocks. Thus, a positive value indicates a higher value than expected when compared to the breed standard at that age, while a minus value indicates a lower value than expected when compared to the breed standard at that age.

**Table 4:** Tests for including and excluding fixed effect terms in the model relating 5% peak egg production duration to physical features of the laying house, hen avoidance behaviours and all stockperson behaviours. Tests for the inclusion of terms for main stockperson behaviours and attitudes of main stockperson had *p* > 0.05, and thus are not presented. All Wald F tests have 1 numerator degrees of freedom. A transformation in parentheses after a term indicates that the variable was examined for inclusion/exclusion in the model after the variable had been transformed, so as to reduce skewness of the variable.

**Terms Included**	**F Value**	**Denominator Degrees of Freedom**	***p*-Value**
Cage Width Grouping (≤50 cm or >60 cm)	118.66	7.0	**0.000012**
Average withdrawal distance in Stroll Test (AvWD)	10.28	7.7	**0.010**
Noise (square root) behaviour for all stockpersons (AllSPNoise)	12.66	7.5	**0.0082**
**Terms Excluded**	**F Value**	**Denominator Degrees of Freedom**	***p*-Value**
Extension of terms in model			
Cage width (included as a variate)	1.08	9.7	0.32
Square of AvWD	0.11	9.9	0.75
Square of Noise	0.12	6.7	0.74
AvWD response differs with cage width grouping	0.43	11.0	0.53
Noise response differs with cage width grouping	0.66	6.9	0.44
Product of AvWD and Noise	0.35	11.0	0.57
Stress physiology			
Albumen corticosterone (logarithm)	0.30	7.2	0.60
Shed features			
Strain of bird	0.00	7.1	0.97
No. of stock people	0.56	6.3	0.48
Age of birds	0.04	7.2	0.84
Flock size	0.12	10.1	0.74
Aisle length	0.26	5.3	0.63
Av. No. of Birds per cage	0.28	7.3	0.62
Space allowance	0.43	7.1	0.53
Cage height	0.85	6.6	0.39
Cage depth	0.01	5.8	0.92
Lux (log(y+1) transformed)	0.03	5.8	0.86
Hen avoidance behaviours			
Proportion of heads out/m in Stroll Test	0.06	6.7	0.82
Forward score in AHT	6.22	5.5	0.051
Heads out score in AHT	0.04	6.6	0.85
All stockperson behaviours			
Approach (square root)	0.06	5.9	0.82
Av SOM	1.73	10.6	0.22
Contact (square root)	0.46	10.9	0.51
Entry (square root)	0.09	5.6	0.78
Handle (square root)	0.02	5.8	0.88
Max SOM	2.43	6.8	0.16
Min SOM	3.14	5.4	0.10
Near Cage (square root)	0.49	10.4	0.50
Stationary (square root)	0.12	5.2	0.74
Time Ends Aisles	0.01	6.3	0.92
Time in Aisle (logarithm)	4.30	8.9	0.068
Time SOH	0.03	6.0	0.86
Total Time (square root)	2.54	9.2	0.15
Visual (square root)	0.82	8.6	0.39

AvWD = Average withdrawal distance, SOM = speed of movement, SOH = start of laying house, AHT = Approaching Human Test. *p*-values less than 0.05 are indicated in bold.

**Table 5:** Tests for including and excluding fixed effect terms in the model relating the logarithm of albumen corticosterone concentration to physical features of the laying house, hen avoidance behaviours and all stockperson behaviours. Tests for the inclusion of terms for the main stockperson behaviours and attitudes of main stockperson had *p* > 0.05, and thus are not presented. Except where indicated, all F tests have 1 numerator degrees of freedom. A transformation in parentheses after a term indicates that the variable was examined for inclusion/exclusion in the model after the variable had been transformed, so as to reduce skewness of the variable.

**Terms Included**	**F Value**	**Denominator Degrees of Freedom**	***p*-Value**
Average withdrawal distance in Stroll Test (AvWD)	13.81	17	**0.0017**
**Terms Excluded**	**F Value**	**Denominator Degrees of Freedom**	***p*-Value**
Extension of terms in model			
Square of AvWD	0.07	16	0.80
Shed features			
Strain of bird (2 denominator degrees of freedom)	0.11	15	0.90
No. of stock people	0.17	16	0.69
Age of birds	1.48	16	0.24
Flock size	0.19	16	0.67
Aisle length	0.65	16	0.43
Cage width	1.11	16	0.31
Av. No. of Birds per cage	0.67	16	0.43
Space allowance	0.21	16	0.66
Cage height	0.03	13	0.87
Cage depth	0.36	15	0.56
Lux (log(y+1) transformed)	0.56	16	0.46
Hen avoidance behaviours			
Proportion of heads out/m in Stroll Test	0.76	16	0.40
Forward score in AHT	0.11	16	0.75
Heads out score in AHT	0.97	16	0.34
All stockperson behaviours			
Approach (square root)	0.25	16	0.62
Av SOM	0.06	16	0.81
Contact (square root)	0.66	16	0.43
Entry (square root)	0.00	16	0.98
Handle (square root)	1.40	16	0.26
Max SOM	0.00	16	0.97
Min SOM	0.01	16	0.93
Near Cage (square root)	0.30	16	0.59
Stationary (square root)	0.22	16	0.64
Time Ends Aisles	0.48	16	0.50
Time in Aisle (logarithm)	0.02	16	0.89
Time SOH	0.10	16	0.75
Total Time (square root)	0.03	16	0.86
Noise (square root)	3.32	16	0.09
Visual (square root)	0.05	16	0.83

AvWD = Average withdrawal distance, SOM = speed of movement, SOH = start of laying house, AHT = Approaching Human Test. *p*-values less than 0.05 are indicated in bold.

**Table 6:** Tests for including and excluding terms in the model relating average withdrawal distance in the Stroll Test to strain of bird, all stockperson behaviours except noise, and attitudes. Tests for the inclusion of terms for main stockperson behaviours gave similar results to those for all stockperson behaviours parameters, but with less residual degrees of freedom, and thus are not presented. Tests for the inclusion of terms for other layer house parameters had *p* > 0.05, and thus are not presented. Except where indicated, all F tests have 1 numerator degrees of freedom. A transformation in parentheses after a term indicates that the variable was examined for inclusion/exclusion in the model after the variable had been transformed, so as to reduce skewness of the variable.

**Terms Included**	**F Value**	**Denominator Degrees of Freedom**	***p*-Value**
Cage Height	12.69	14	**0.0031**
**Terms Excluded**	**F Value**	**Denominator Degrees of Freedom**	***p*-Value**
Extension of terms in model			
Square of Cage Height	0.03	13	0.88
Strain of bird			
Strain of bird (2 numerator degrees of freedom)	1.46	2, 12	0.27
All stockperson behaviours			
Approach (square root)	0.01	13	0.91
Av SOM	2.37	13	0.15
Contact (square root)	3.92	13	0.069
Entry (square root)	0.37	13	0.55
Handle (square root)	1.51	13	0.24
Max SOM	0.58	13	0.46
Min SOM	0.13	13	0.73
Near Cage (square root)	3.09	13	0.10
Stationary (square root)	1.98	13	0.18
Time Ends Aisles	5.22	13	**0.040**
Time in Aisle (logarithm)	0.31	13	0.59
Time SOH	0.03	13	0.88
Total Time (square root)	0.35	13	0.56
Noise (square root)	0.20	13	0.66
Visual (square root)	0.69	13	0.42
Main stockperson attitudes			
Empathy	0.18	9	0.68
Sensitivity of stockperson	0.00	9	0.95
Insensitivity of stockperson	4.98	9	0.053
Job control	1.14	9	0.31
Stockperson enjoys job	1.13	9	0.32
Diligence	0.00	9	0.96
No job control	1.14	9	0.31
Unpleasantness of job	0.47	9	0.51
Negative general attitudes	1.38	9	0.27
Positive general attitudes	1.13	9	0.32

SOM = speed of movement, SOH = start of laying house. *p*-values less than 0.05 are indicated in bold.

**Table 7:** Tests for including and excluding terms in the model relating main stockperson noise (square root transformed) to strain of bird, all stockperson behaviours except noise and attitudes. Tests for the inclusion of terms for other physical features of the laying house, main stockperson behaviours had *p* > 0.1, and thus are not presented. Except where indicated, all F tests have 1 numerator degrees of freedom.

**Terms Included**	**Wald F Value**	**Denominator Degrees of Freedom**	***p*-Value**
All stockperson entry behaviour (square root) (AllSPEntry)	25.34	10	**0.00051**
Main stockperson positive attitude score (MainPositive)	14.96	10	**0.0031**
Main stockperson insensitivity attitude score (MainInsensitivity)	10.68	10	**0.0085**
**Terms Included**	**Wald F Value**	**Denominator Degrees of Freedom**	***p*-Value**
Extension of terms in model			
Square of AllSPEntry	0.11	9	0.75
Square of MainSPPositive	0.43	9	0.53
Square of MainSPInsensitivity	3.06	9	0.11
Product of AllEntry and MainSPPositive	0.32	9	0.59
Product of AllEntry and MainInsensitivity	0.08	9	0.78
Product of MainPositive and MainInsensitivity	0.00	9	0.95
Strain of bird			
Strain of bird (2 numerator degrees of freedom)	5.57	8	0.031
Strain of bird (after deleting a single laying house with Ingham Hisex)	2.13	8	0.18
All stockperson behaviours			
Approach (square root)	0.87	8	0.38
Av SOM	0.87	8	0.38
Contact (square root)	0.00	8	0.95
Handle (square root)	0.02	8	0.89
Max SOM	0.24	8	0.64
Min SOM	0.59	8	0.46
Near Cage (square root)	0.04	8	0.85
Stationary (square root)	0.72	8	0.42
Time Ends Aisles	0.04	8	0.84
Time in Aisle (logarithm)	0.00	8	0.95
Time SOH	0.22	8	0.65
Total Time (square root)	0.02	8	0.90
Visual (square root)	0.07	8	0.80
Main stockperson attitudes			
Empathy	2.08	9	0.18
Sensitivity of stockperson	0.07	9	0.79
Job control	0.36	9	0.56
Stockperson enjoys job	1.27	9	0.29
Diligence	3.69	9	0.087
No job control	0.99	9	0.35
Unpleasantness of job	0.26	9	0.62
Negative general attitudes	0.01	9	0.94

SOM = speed of movement, SOH = start of laying house. *p*-values less than 0.05 are indicated in bold.
